# Supplementary material for: Alpha-synuclein misfolding as fluid biomarker for Parkinson’s disease measured with the iRS platform
Source: EMBO Mol Med. 2025 Apr 25;17(6):1203–21. doi: 10.1038/s44321-025-00229-z (PMC12162852; doi:10.1038/s44321-025-00229-z)
Supplement: Supplementary file 1 — Appendix [file 44321_2025_229_MOESM1_ESM.pdf]

Appendix

Alpha-synuclein misfolding as fluid biomarker for Parkinson's disease measured with the iRS platform

Martin Schuler[1,2], Grischa Gerwert[1,2], Marvin Mann[1,2], Nathalie Woitzik[1,2], Lennart Langenhoff[1,2], Diana Hubert[1,2], Deniz Duman[1,2], Adrian Höveler[1,2], Sandy Galkowski[1,2], Jonas Simon[1,2], Robin Denz[3], Sandrina Weber[4], Eun-Hae Kwon[5], Robin Wanka[1,2], Carsten Kötting[1,2], Jörn Güldenhaupt[1,2], Léon Beyer[1,2], Lars Tönges[5], Brit Mollenhauer[4,6-7], Klaus Gerwert[1,2]

(1) Center for Protein Diagnostics (PRODI), Ruhr-University Bochum, Bochum, Germany
(2) Department of Biophysics, Ruhr-University Bochum, Bochum, Germany
(3) Department of Medical Informatics, Biometry and Epidemiology, Ruhr-University Bochum, Germany
(4) University Medical Center Göttingen, Department of Neurology, Göttingen, Germany
(5) Department of Neurology, St. Josef-Hospital, Ruhr-University Bochum, Bochum, Germany
(6) Paracelsus-Elena-Klinik, Kassel, Germany (Klinikstraße 16, 34128 Kassel, Germany)
(7) Scientific employee with an honorary contract at Deutsches Zentrum für Neurodegenerative Erkrankungen (DZNE), Göttingen, Germany.

Appendix Figure	Description	Page
S1	Indirect ELISA for EC_{50}-value determination of native and labeled capture antibody	2
S2	Logistic regression models for the combined dataset.	3
S3	Reproducibility measures with a pooled control CSF sample measured on the αSyn capture antibody surface	4
S4	Dataset distribution and conformity to normal distribution by normal Q-Q-Plot of 1656.0/1623.5-Ratios	5

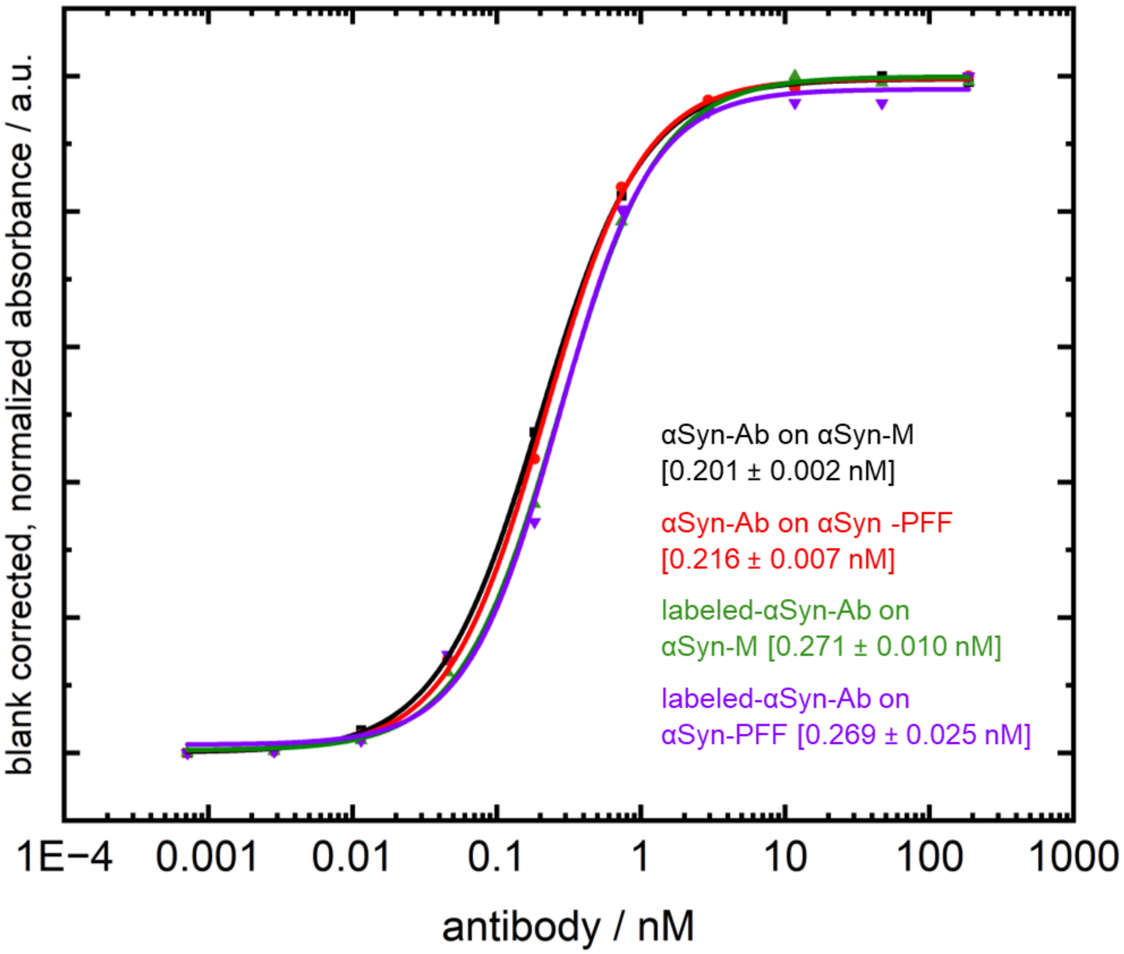

Appendix Figure S1: Indirect ELISA for EC_{50}-value determination of native and labeled capture antibody according to given protocol (EV Materials). EC_{50}-values were determined from blank corrected absorbance difference at 450 - 620nm using a 4-parameter logistic fit model on normalized data. Values demonstrate the fit results of the native and labeled antibody towards αSyn-M (Stressmarq Bioscience INC. SPR-321) and αSyn-PFF (Stressmarq Bioscience INC. SPR-322), as used in the ThT and ATR-FTIR experiments. EC_{50} values from the 4-parameter logistic fit model are 0.20 ± 0.002 nM and 0.22 ± 0.007 nM for native antibody, while labeled antibody shows EC_{50} values of 0.27 ± 0.010 nM and 0.27 ± 0.025 nM.

	Demographics			Demographics + iRS		
**Characteristic**	**OR[1]**	**95% CI[1]**	**p-value**	**OR[1]**	**95% CI[1]**	**p-value**
Age	1.020	0.988, 1.055	0.2	1.027	0.986, 1.076	0.2
Gender						
m	—	—		—	—	
w	0.264	0.119, 0.564	$7.5 \cdot 10^{-4}$	0.298	0.105, 0.817	0.020
readout10				0.030	0.006, 0.101	$5.6 \cdot 10^{-7}$

[1]OR = Odds Ratio, CI = Confidence Interval

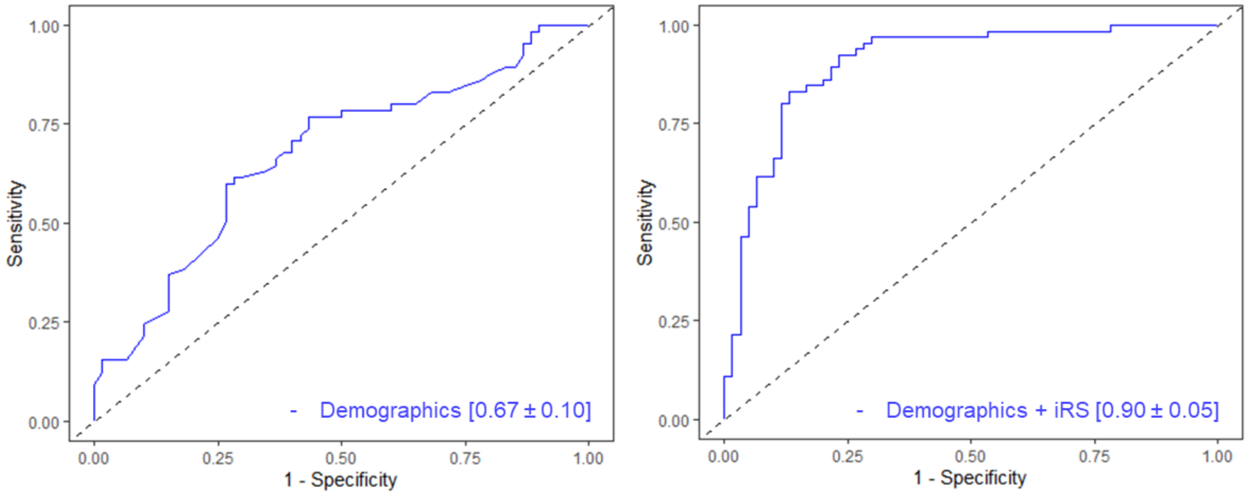

Appendix Figure S2: Logistic regression models for the combined dataset. The first model, "Demographics" accounts for age and sex only, while the second model "Demographics + iRS," also accounts for the iRS assay read-out. The clinical diagnosis is always used as the dependent variable. Calculated models with characteristics in the upper table were utilized to calculate ROC-AUC curves for both models. The demographics-only model showed an AUC of 0.67 ± 0.10 (p-value $7.5 \cdot 10^{-4}$), while with additional iRS read-out, the AUC increased to 0.90 ± 0.05 (p-value $5.6 \cdot 10^{-7}$). The shown p-values were calculated using a standard Wald test.

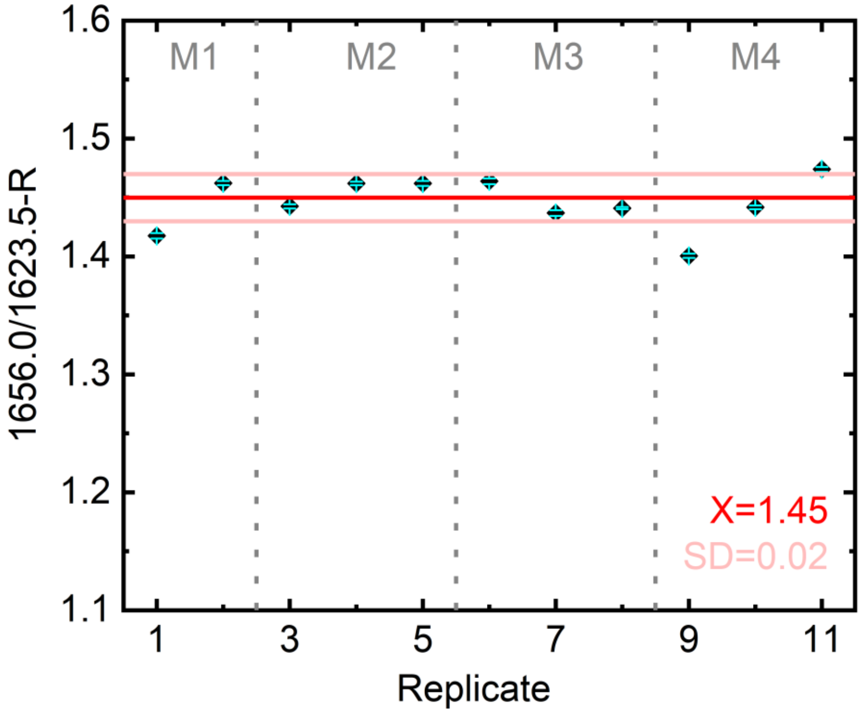

Appendix Figure S3: Reproducibility measures with a pooled control CSF sample measured on the αSyn capture antibody surface. The eleven replicates were measured in four measurements (M1-M4). Routine spectra processing (WV and baseline correction, averaging spectra and reference channel subtraction, Fourier self-deconvolution (FSD), and smoothing) was performed. Each replicate is depicted with an error bar (cyan) obtained from applying a smoothing variation by the in-house MATLAB script for data analysis. The mean 1656.0/1623.5-ratio of all measurements was $\bar{X}$=1.45 with a standard deviation of SD=0.02.

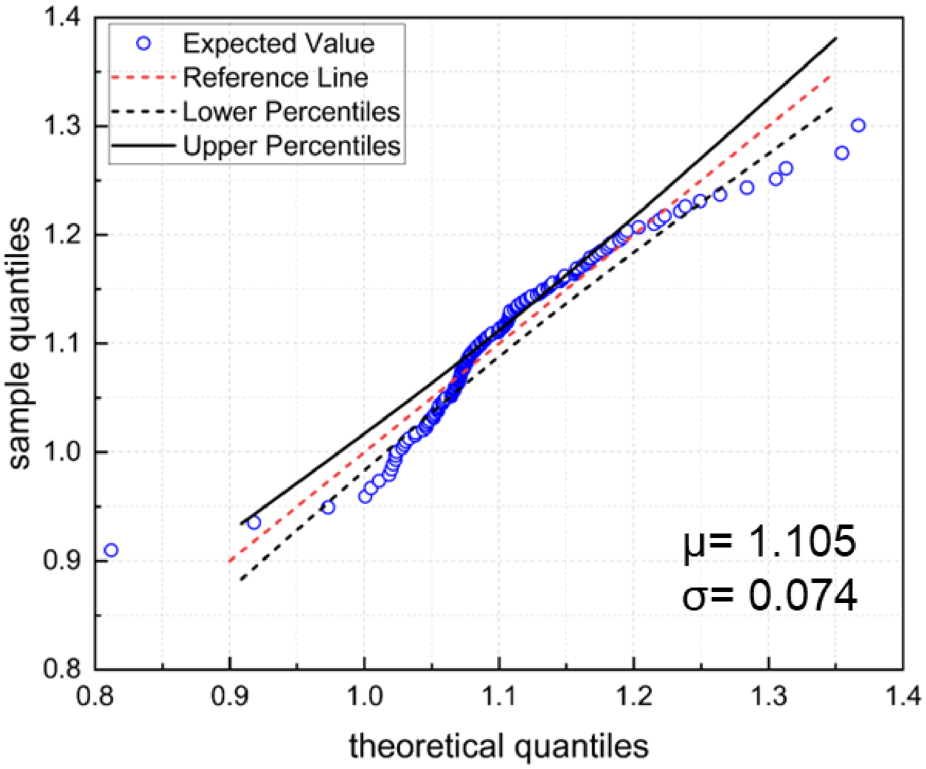

Appendix Figure S4: Dataset distribution and conformity to normal distribution by normal Q-Q-Plot of 1656.0/1623.5-Ratios. The mean of the normal distribution is μ=1.105 with a standard deviation of σ=0.074 for the normal distribution. Lower and upper percentiles (black line and dashed black line) mark 95 %-confidence bands, while the red dashed line shows the reference line. Since a considerable amount of data points of the combined data set (n=134) are not located within the confidence borders, the dataset is not normally distributed. Thus, non-parametric models were applied to test the significance of group separations.
