# Supplementary material for: Alpha-synuclein misfolding as fluid biomarker for Parkinson’s disease measured with the iRS platform
Source: EMBO Mol Med. 2025 Apr 25;17(6):1203–21. doi: 10.1038/s44321-025-00229-z (PMC12162852; doi:10.1038/s44321-025-00229-z)
Supplement: Supplementary file 8 — Expanded View Figures [file 44321_2025_229_MOESM8_ESM.pdf]

Expanded View Figures

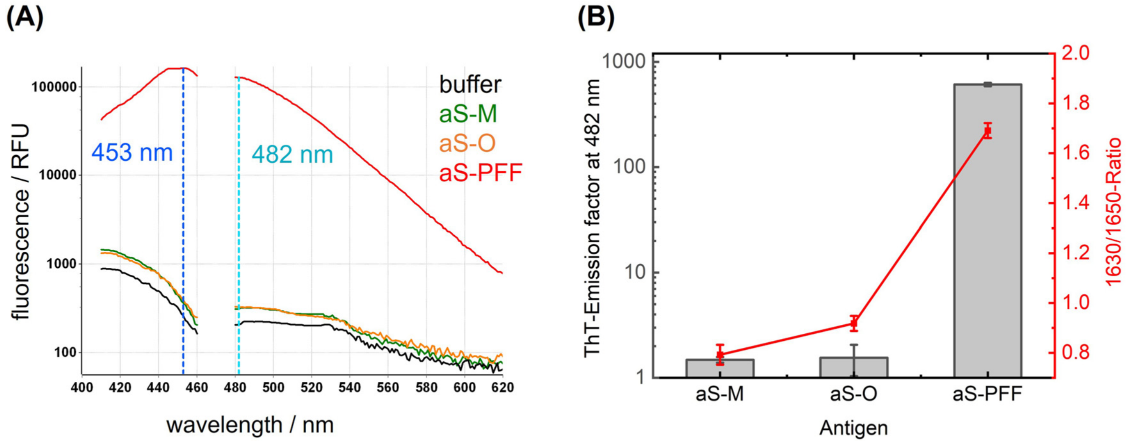

Figure EV1. ThT analysis of ∝Syn antigens.

A The mean ThT-excitation and emission spectra from sample triplicates, recorded from 410–460 nm and 480–620 nm and displayed in relative fluorescence units (RFUs). For this, technical triplicates of αSyn-M (Stressmarq Bioscience Inc., SPR-321), αSyn-O (Stressmarq Bioscience Inc., SPR-466), and αSyn-PFFs (Stressmarq Bioscience Inc., SPR-322) were prepared with a final concentration of 7.14 µM in 25 µM ThT-PBS buffer, incubated for 60 min at ambient temperature in the 96 F-bottom fluorescence plate and measured with the plate reader in spectral scan mode. The excitation scan was performed from 410–460 nm, while the emission scan was performed from 480–620 nm, with a bandwidth of 10 nm and 20 flashes/well. The gain was adjusted at expected maxima (453/482 nm), and autofocus obtained focal height. Before read-out, the plate was shaken at 300 rpm for 5 s. (**B**) Mean ThT intensities at 482 nm were calculated from triplicate ThT measures of the respective antigens. The ThT-emission factor was calculated with mean intensities at 482 nm by division of mean intensities of the buffer at 482 nm (25 µM ThT-PBS). Moreover, the 1630/1650-R, derived from ATR-FTIR measurement in duplicates, was plotted against the ThT factor since the increased 1630/1650-R shows increased ß-sheet content of the given protein and decreased α-helical/random-coil content. To obtain the 1630/1650-R without the influence of binding tendencies of antibodies, antigens were measured directly on the Bruker alpha ATR-FTIR cell against the respective buffer (PBS, pH 7.4). The 1650/1630-R was retrieved automatically by the same MATLAB script used for the CSF samples after averaging 10 sample spectra for increased S/N. Error bars display the standard deviation of the ATR-duplicates or ThT-triplicates.

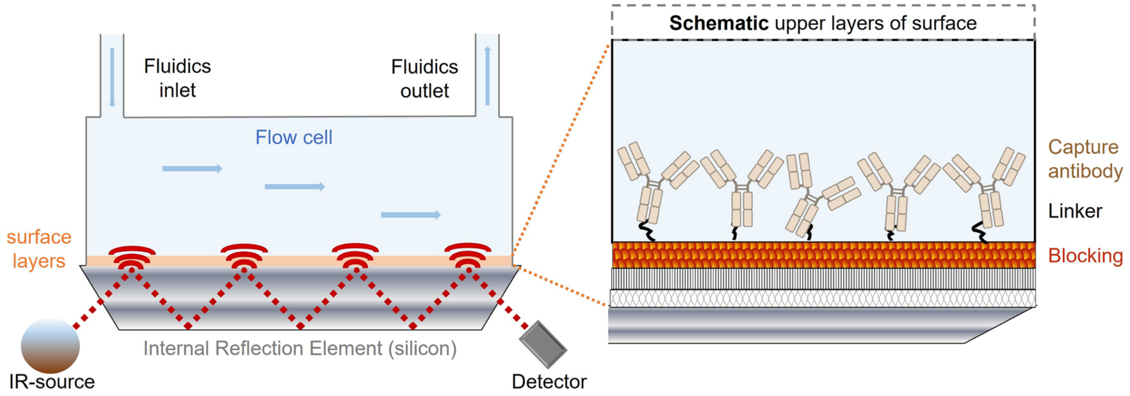

Figure EV2. Scheme of immuno-infrared sensor (iRS).

The concept was published before in detail ((Nabers et al, 2016a, 2016c, 2018; Schartner et al, 2013)). The body fluid sample (without additives or modifications) is circulated in the sample chamber over the functionalized crystal (internal reflection element) surface in an ATR (Attenuated Total Reflection) setup ((**A**); image not to scale). At points of total reflection, an evanescent field (red) is generated invading only about 500 nm and preventing large water absorbance in the amide-I region (Goormaghtigh et al, 1990). The area of the evanescent field marks the measurable area in the iRS setup. (**B**) (image not to scale): The approach for surface functionalization is described in full detail (WO2024003213A1 and WO2024003214A3). The surface of the internal reflection element is functionalized with a capture antibody, which extracts all αSyn conformers from the body fluid. The antibody is bound by a linker to a blocking layer on the surface of the iRS. The blocking layer prevents unspecific binding on the surface of the iRS. The evanescent infrared beam measures the absorbance maximum of the amide-I band as read out. The αSyn absorbance is then elucidated by difference spectroscopy. For this, a background reference spectrum is taken before sample application and subtracted as background reference. The difference spectrum reveals the absorbance spectrum of all bound αSyn conformers without background from prior surface layers due to functionalization and water. Depending on the distributions of the conformers, the read-out varies between ~1655 cm^{-1} wavenumbers for a pure α-helical/random-coil distribution and ~1625 cm^{-1} for a pure β-sheet conformer distribution. Mixtures absorb in between depending on the conformer distribution. The downshift of the read-out represents the disease progression in a continuum by the secondary structure distribution of αSyn.

 © The Author(s)

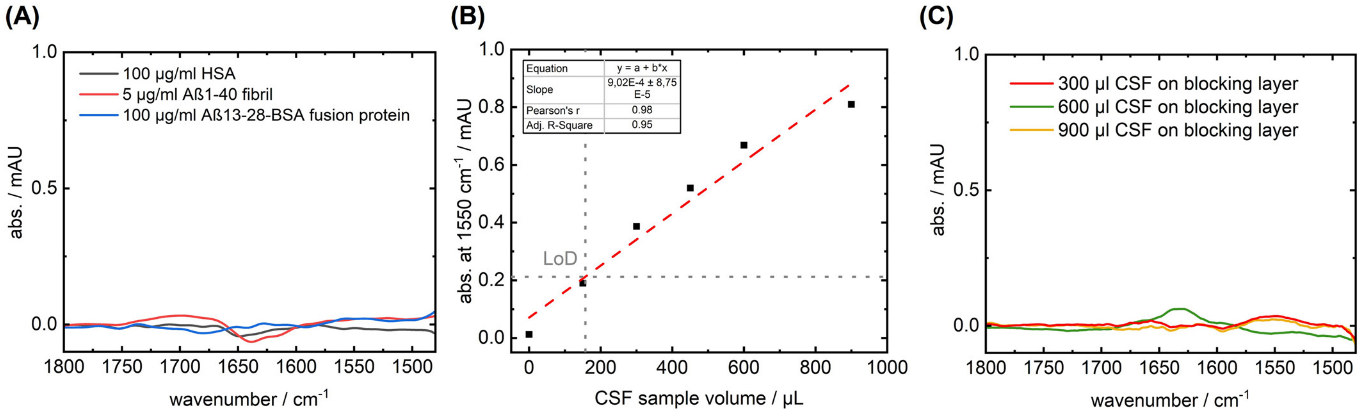

Figure EV3. Testing of unspecific binding to the blocking layer.

(**A**) The cross-reactivity test by mean wash spectra in PBS on an αSyn -capture antibody surface with HSA, Aß$_{1–40}$-fibrils, and Aß$_{13–28}$-BSA fusion protein (produced by two-step sulfo-NHS reaction with primary amines; Thermo Fisher Scientific™, art. no. 39269) in 5–100 µg range in 1 ml total volume. No bound signal is observed in all cases; thus, no cross-reactivity to abundant HSA and other amyloidogenic proteins such as β-amyloid in the form of a ß-amyloid$_{1–40}$ fibril (preparation according to Shin et al) or conjugated β-amyloid$_{13–28}$ (monomeric form) in high concentrations is observed (Shin et al, 1997). (**B**) The amount of immobilized protein on an αSyn capture antibody surface by absorbance at 1550 cm^{-1} in relation to the used CSF volume. The assay signal between 300–900 µl shows alignment to linear fit, implying enough capture antibody molecules for the target analyte as expected for antibody excess surfaces. The limit of detection (LoD) was calculated by LoD = 3.3 (σ/s) with σ = standard deviation and s = slope / coefficient of X-variable to LoD = 3.3·(0.043/9.02·10^{-4}) = 157 µl CSF. Since S/N is critical when determining the secondary structure-sensitive amide-I spectral features and the aSyn amount in samples vary naturally, CSF volumes of 300 µl were analyzed for each sample. (**C**) The inertness of the blocking layer without the capture antibody on the surface for 300–900 µl CSF sample volume as mean wash spectra in PBS. No specific signals were observed at 1550 cm^{-1} (= bound protein) or in the amide-I region (1600–1700 cm^{-1}) without the capture antibody on the assay's blocking layer.

© The Author(s)

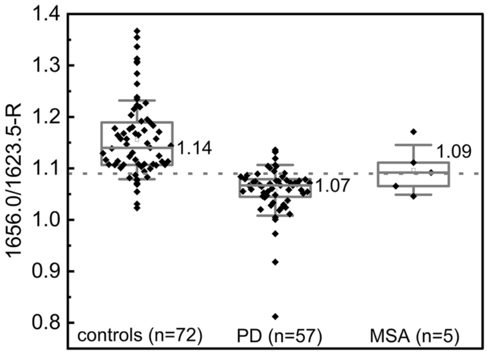

Figure EV4. Subgroup comparison of PD and MSA samples to controls.

The five presented MSA cases are closer to controls than PD subjects regarding the iRS-readout measuring the αSyn misfolding. The two synucleinopathies, MSA and PD, are overlapping, although the number of MSA cases is still not significant yet. Box and whisker plots show median value (vertical line), interquartile range (boxes), and 1x standard deviation (whisker), while the given values report the median value of the groups. The box minima and maxima are 1.08/1.23 (controls), 1.01/1.11 (PD), and 1.05/1.15 (MSA). Note: small and unbalanced datasets in the case of MSA hinder robust interpretation of group differences.

 © The Author(s)

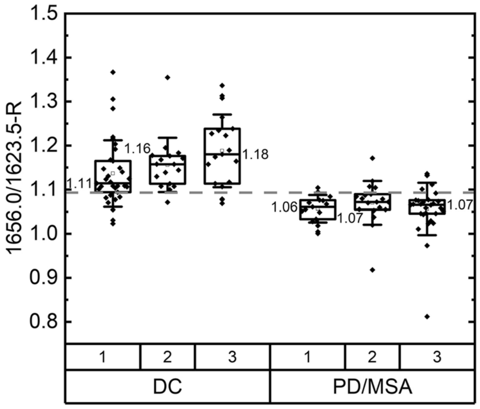

M.-W. U: exact p-values	PM1 (n=17)	PM2 (n=19)	PM3 (n=29)
DC1 (n=33)	$1.5\cdot10^{-6}$ (****)	$1.8\cdot10^{-4}$ (***)	$9.9\cdot10^{-7}$ (****)
DC2 (n=17)	$3.9\cdot10^{-8}$ (****)	$4.1\cdot10^{-6}$ (****)	$3.2\cdot10^{-8}$ (****)
DC3 (n=19)	$1.6\cdot10^{-7}$ (****)	$9.1\cdot10^{-6}$ (****)	$9.1\cdot10^{-9}$ (****)

Figure EV5. Sample distribution ($n = 134$) on three independent iRS instruments (1,2,3).

Different devices with randomized sample sets are comparable, and separation is highly significant. The whiskers of Boxplots depict 1× standard deviation, while the given values report the median value of the groups. The box minima and maxima are 1.06/1.21 (DC, 1), 1.09/1.22 (DC, 2), 1.11/1.27 (DC,3), 1.03/1.09 (PD/MSA, 1), 1.02/1.12 (PD/MSA, 2), and 1.00/1.12 (PD/MSA, 3) All subgroup comparisons by Mann–Whitney U testing are listed in the adjacent table (p values between $1.8\cdot10^{-4}$ to $9.1\cdot10^{-9}$).
